# Acknowledgement of manuscript reviewers 2015

**DOI:** 10.1186/s13011-016-0054-5

**Published:** 2016-02-29

**Authors:** Stephan Arndt, Janet Zwick

**Affiliations:** University of Iowa, Iowa City, USA; Iowa Department of Public Health, Des Moines, Iowa, USA

## Abstract

The editors of *Substance Abuse Treatment, Prevention, and Policy* would like to thank all our reviewers who have contributed to the journal in Volume 10 (2015).

**Laura Acion**

Argentina

**Farrokh Alemi**

USA

**Jon-Patrick Allem**

USA

**F Javier Alvarez**

Spain

**Chaisiri Angkurawaranon**

Thailand

**Colin Angus**

UK

**Richmond Aryeetey**

Ghana

**Vassilis Barkoukis**

Greece

**Eric Benotsch**

USA

**Arvin Bhana**

South Africa

**Eva Biringer**

Norway

**Cornelia Blank**

Austria

**Terry Blum**

USA

**Ricky N Bluthenthal**

USA

**Ian Boardley**

UK

**Udo Bonnet**

Germany

**Alain Braillon**

France

**Ralf Brand**

Germany

**Daniel Bressington**

Hong Kong

**Janice Brown**

USA

**Alan Budney**

USA

**William Burk**

Netherlands

**John Burns**

UK

**Andreas Büttner**

Germany

**Jane A Buxton**

Canada

**Tom Byrne**

USA

**Catherine Canamar**

USA

**Adrian Carter**

Australia

**Tomas Cartmill**

Australia

**Sari Castrén**

Finland

**Alan Cavaiola**

USA

**Timmen Cermak**

USA

**Emeline Chauchard**

France

**Tessa Cheng**

Canada

**Sandhya Cherkil**

India

**Neil Chester**

UK

**Theodore J Cicero**

USA

**Penny Cook**

UK

**Stephanie Cook**

USA

**Claudia Costa Storti**

Portugal

**Simon Coulton**

UK

**Cheryl Currie**

Canada

**Berihun Dachew**

Ethiopia

**Cassandra Davis**

Australia

**Olivier De Hon**

Netherlands

**Paolo Deluca**

UK

**Zsolt Demetrovics**

Hungary

**Bryan Denham**

USA

**Anahi Dreser**

Mexico

**Maria Duaso**

UK

**Matthew Dunn**

Australia

**Debra Dwyer**

USA

**Lindsey M Eldred Kozecke**

USA

**Michele Eliason**

USA

**Sarah Elison**

UK

**Kathy Etz**

USA

**Jamison D Fargo**

USA

**James Fell**

USA

**Annesa Flentje**

USA

**Pilar Flores**

Spain

**James Foulds**

New Zealand

**John R Gallagher**

USA

**Casey Gallimore**

USA

**Ewenat Gebrehanna**

Ethiopia

**Michelle Geier**

USA

**Christopher L German**

USA

**Gail Gilchrist**

UK

**Elena Gomes De Matos**

Germany

**Christine Goodall**

UK

**Sean Grant**

USA

**Margaret Griffin**

USA

**Erick Guerrero**

USA

**Xiamei Guo**

China

**Nicholas E Hagemeier**

USA

**Wayne Hall**

Australia

**Nobuyuki Hamajima**

Japan

**Hendrika L Hattingh**

Australia

**Nabila He**

China

**Bryan Heckman**

USA

**Eric Hirsch**

USA

**Elizabeth Hughes**

UK

**Sabrina Anne Jacob**

Malaysia

**Marie P Jauffret-Roustide**

France

**Marianne Jauncey**

Australia

**Lauren Jelenchick**

USA

**Syeda Kanwal**

Pakistan

**Brian Kelly**

USA

**Thomas Kerr**

Canada

**Jason Kilmer**

USA

**Albert Kopak**

USA

**Jeffrey Korte**

USA

**Sarah Krier**

USA

**Joachim Kugler**

Germany

**Magdalena Kulesza**

USA

**Caroline Kuo**

USA

**Jessica Lacroix**

USA

**Wayne Lehman**

USA

**Kathleen Lenk**

USA

**Jenepher Lennox Terrion**

Canada

**Seunghoo Lim**

USA

**Marianne L Lindahl**

Sweden

**Melissa Little**

USA

**Ya-Ming Liu**

Taiwan

**Michael Livingston**

Australia

**Andreas Lundin**

Sweden

**Tara Lyons**

Canada

**Tim K Mackey**

USA

**Kara Macleod**

USA

**Kwok-Kei Mak**

Hong Kong

**Rodrigo Marin-Navarrete**

Mexico

**Michael Martin**

Thailand

**Miesha Marzell**

USA

**Holly Mash**

USA

**Elizabeth Mcgill**

UK

**Erin Mead**

USA

**Lisa R Metsch**

USA

**Sidy Mohamed Seck**

Senegal

**Sabrina Molinaro**

Italy

**Gila Neta**

USA

**Anne M Neumann**

USA

**Dorothy Newbury-Birch**

UK

**Heid Nokleby**

Norway

**Diana Obando**

USA

**Amy O’Donnell**

UK

**David Otiashvili**

Georgia

**Eugenia Oviedo-Joekes**

Canada

**Howard Padwa**

USA

**Nilesh B Patel**

Kenya

**David Patterson Silver Wolf (Adelv Unegv Waya)**

USA

**Cavalcanti Pereira**

Brazil

**Jessica Perkins**

USA

**Andrea Petroczi**

UK

**Rose Pignataro**

USA

**Bill Ponicki**

USA

**Harrison Pope**

USA

**Jenessa Price**

USA

**Robin C Purshouse**

UK

**Ravindra Rao**

India

**Carla J Rash**

USA

**Allecia Reid**

USA

**Kelly Riesenmy**

USA

**Darve Robinson**

USA

**Mark Robinson**

UK

**Jason Robinson**

USA

**Paul Roman**

USA

**Ali Roohbakhsh**

Iran

**Perrine Roux**

France

**Heiko Rueger**

Germany

**Beth Rutkowski**

USA

**Dominic Sagoe**

Norway

**Ethan Sahker**

USA

**Allison Salmon**

Australia

**Albert Sanchez-Niubo**

Spain

**Matej Sande**

Slovenia

**M Santos Cruz**

Brazil

**Enisha Sarin**

India

**Michael Schaefer**

Germany

**Kimberly Schoonover**

USA

**Denise Scott**

USA

**Kashif Shafique**

UK

**Kamaludin Sheikh**

Saudi Arabia

**Marianna Shershneva**

USA

**Stacey Sigmon**

USA

**Janie Simmons**

USA

**Kurt Skarberg**

Sweden

**Samir S Soneji**

USA

**Debra Srebnik**

USA

**Chandrashekhar Sreeramareddy**

Nepal

**Laura Stevens**

Belgium

**Susan A Stoner**

USA

**Carol Strike**

Canada

**Pierluigi Struzzo**

Italy

**Elizabeth Stuyt**

USA

**Josep M Suelves**

Spain

**Nopporn Tantirangsee**

Thailand

**Teeranee Techasrivichien**

Thailand

**Birgitte Thylstrup**

Denmark

**Marelign Tilahun**

Ethiopia

**Alexander Tomei**

Switzerland

**Carla Treloar**

Australia

**Joan Trujols**

Spain

**Ambros Uchtenhagen**

Switzerland

**Jaap Van Der Stel**

Netherlands

**Eleni Vangeli**

UK

**Srinivasaraghavan Venkatesh**

India

**Corrado Villella**

Italy

**Jason Wallach**

USA

**Sara Wallhed Finn**

Sweden

**Jeffrey Wardell**

Canada

**Marta Welbel**

Poland

**Lisa Whitaker**

UK

**Rachel Widome**

USA

**Katharina Wiest**

USA

**T Cameron Wild**

Canada

**Wanja Wolff**

Germany

**Jennifer Wray**

USA

**Zhuo Yang**

USA

**Xibiao Ye**

Canada

**Lance Brendan Young**

USA

**Wenhua Zhou**

China

